# Annual and Analytical Cyclopædia of Practical Medicine

**Published:** 1901-12

**Authors:** 


**Annual and Analytical Cyclopædia of Practical Medicine.** By Charles E. de M. Sajous, M.D., and one hundred associate Editors. Vol. VI. Pp. viii., 1043. Philadelphia: F. A. Davis Company. 1901.

This volume is the last of the first series of this work. It is claimed that the completed work contains more than was promised, and a perusal of the general index of 108 double-column pages shows what a fund of information the six volumes contain. The articles commence with Rectum and Anus, concluding with Zinc. Some of the most notable are on rheumatism, diseases of the stomach, syphilis, surgery of the urinary system, and diseases of the uterus, all written by well-known authorities. The article on typhoid fever, by Dr. James E. Graham, of Toronto, is very complete, extending to 36 double pages. That on valvular diseases of the heart and endocarditis extends to 18 pages, by Dr. Herman F. Vickery, of Boston. It is impossible to speak too highly of the contents of these six volumes and of their mounting. The type is large enough for easy reading; there is no useless padding; there is a liberal diffusion of chromo-lithographic engravings and maps, the whole giving quite an encyclopædia of practical medicine which may well take the place of all others.

